# Radiographic remission in rheumatoid arthritis quantified by computer-aided joint space analysis (CASJA): a post hoc analysis of the RAPID 1 trial

**DOI:** 10.1186/s13075-020-02322-9

**Published:** 2020-10-06

**Authors:** Alexander Pfeil, Anica Nussbaum, Diane M. Renz, Tobias Hoffmann, Ansgar Malich, Marcus Franz, Peter Oelzner, Gunter Wolf, Joachim Böttcher

**Affiliations:** 1 Department of Internal Medicine III, Jena University Hospital – Friedrich Schiller University Jena, Am Klinikum 1, 07747 Jena, Germany; 2 Institute of Diagnostic and Interventional Radiology, Department of Pediatric Radiology, Hannover Medical School, Carl-Neuberg-Str. 1, 30625 Hannover, Germany; 3 Institute of Diagnostic Radiology, Suedharz-Hospital Nordhausen, Dr. Robert-Koch-Straße 38, 99734 Nordhausen, Germany; 4 Department of Internal Medicine I, Jena University Hospital – Friedrich Schiller University Jena, Am Klinikum 1, 07747 Jena, Germany; 5 Faculty of Medicine, Jena University Hospital – Friedrich Schiller, University Jena, Am Klinikum 1, 07747 Jena, Germany; 6 Institute of Diagnostic and Interventional Radiology, Jena University Hospital – Friedrich Schiller, University Jena, Am Klinikum 1, 07747 Jena, Germany

**Keywords:** Rheumatoid arthritis, RA, Computer-aided joint space, CASJA, Joint space width, JSW, Joint space distance, JSD, Certolizumab pegol, Methotrexate, MTX, Radiographic remission

## Abstract

**Background:**

The reduction of finger joint space width (JSW) in patients with rheumatoid arthritis (RA) is strongly associated with joint destruction. Treatment with certolizumab pegol (CZP), a PEGylated anti-TNF, has been proven to be effective in RA patients. The computer-aided joint space analysis (CAJSA) provides the semiautomated measurement of joint space width at the metacarpal-phalangeal joints (MCP) based on hand radiographs. The aim of this post hoc analysis of the RAPID 1 trial was to quantify MCP joint space distance (JSD-MCP) measured by CAJSA between baseline and week 52 in RA patients treated with certolizumab pegol (CZP) plus methotrexate (MTX) compared with MTX/placebo.

**Methods:**

Three hundred twenty-eight patients were included in the post hoc analysis and received placebo plus MTX, CZP 200 mg plus MTX and CZP 400 mg plus MTX. All patients underwent X-rays of the hand at baseline and week 52 as well as assessment of finger joint space narrowing of the MCP using CAJSA (Version 1.3.6; Sectra; Sweden). The joint space width (JSW) was expressed as mean joint space distance of the MCP joints I to V (JSD-MCP_total_).

**Results:**

The MTX group showed a significant reduction of joint space of − 4.8% (JSD-MCP_total_), whereas in patients treated with CZP 200 mg/MTX and CZP 400 mg/MTX a non-significant change (JSD-MCP_total_ + 0.6%) was observed. Over 52 weeks, participants with DAS28 remission (DAS28 ≤ 2.6) exhibited a significant joint space increase of + 3.3% (CZP 200 mg plus MTX) and + 3.9% (CZP pegol 400 mg plus MTX).

**Conclusion:**

CZP plus MTX did not reduce JSD-MCP_total_ estimated by CAJSA compared with MTX/placebo. Furthermore, clinical remission (DAS28 ≤ 2.6) in patients treated with CZP plus MTX was associated with an increasing JSD, indicating radiographic remission in RA.

## Key message

- Treatment with certolizumab pegol is associated with the absence of joint space narrowing as detected by computer-aided joint space analysis (CASJA).
- Clinical remission (DAS28 ≤ 2.6) is associated with finger joint space widening in RA patients treated with certolizumab pegol.
- Clinical remission in combination with finger joint space widening represents radiological remission in RA.
- The improvement of HAQ-DI is related to an increased finger joint space width which corresponded with an improved joint function.

## Background

Rheumatoid arthritis (RA) is a chronic inflammatory disease characterized by persistent synovitis which ultimately leads to the destruction of articular structure and subchondral bone [1]. The joints involved most frequently are the metacarpophalangeal (MCP) joints of the hands, the wrists and small joints of the feet. In established RA, radiographs reveal joint space narrowing as a result of cartilage loss and characteristic erosions of the bone.

Currently, radiographic scoring systems, especially the modified Sharp*/*van der Heijde scoring (SHS), are the gold standard for the evaluation of joint space width (JSW) in studies and clinical routine [2]. Due to the introduction of effective RA therapy strategies such as biological and targeted synthetic disease-modifying antirheumatic drugs (DMARDs), traditional scoring methods may fail to detect differences of radiographic progression between treatment and placebo groups [3].

In the last years, different computer-based techniques were developed to assess finger JSW using hand radiographs [4–6]. One of these methods is the new computer-aided joint space analysis (CAJSA, Version 1.3.6; Sectra; Sweden), which conducts semi-automated measurements of joint space distances (JSD) at the metacarpal-phalangeal joints (MCP) by using digitized hand radiographs. In recent studies, CAJSA has been reported to detect and quantify joint space narrowing of MCP as typical RA-related radiographic changes [7–9]. In addition, the CAJSA-technique was able to differentiate between treatment groups, whereas the Sharp Score did not show any significant changes [9]. The superior performance of CAJSA is based on a high reproducibility (interradiographic reproducibility 0.63% and intraradiographic reproducibility 0.38% for digital X-rays) [10] as well as a high sensitivity of 88.1% and specificity of 77.8% for the measurement of joint space narrowing in RA [8].

The aim of this post hoc analysis based of the RAPID (Rheumatoid Arthritis Prevention of Structural Damage) 1 trial [11] was the quantification of joint space distance (JSD) of MCP (JSD-MCP) measured by CAJSA between baseline and week 52 for the placebo/methotrexate (MTX) group compared to certolizumab pegol (CZP) 200 mg plus MTX and CZP 400 mg plus MTX. Additionally, the JSD-MCP values were evaluated in patients with clinical remission (Disease Activity Score {DAS} 28 ≤ 2.6) and without clinical remission (DAS28 > 2.6) under the treatment with CZP 200 mg/MTX versus CZP 400 mg/MTX.

## Methods

### Study population

The post hoc analysis encompassed a total of 328 RA patients included in the 52-week, phase III, multicentre, randomized, double-blind, placebo-controlled, parallel-group trial RAPID 1 trial [11]. Patients had received placebo plus MTX (*N* = 31), CZP 200 mg plus MTX (*N* = 149) or CZP 400 mg plus MTX (*N* = 148).

Radiographs of the hands (anteroposterior) and feet (posteroanterior) were obtained at baseline and week 52. The images have been checked regarding quality as described by Pfeil et al. [12], and 17 patients were excluded because of not measurable JSW by CASJA. Clinical remission was assessed based on DAS28 (DAS28 ≤ 2.6) [13].

### Measurement of JSW by CASJA

All subjects underwent measurements of the JSD at the MCP joints (thumb to small finger; JSD-MCP) using the CAJSA technology (Radiogrammetry Kit, Version 1.3.6; Sectra; Sweden). The measurement started with the positioning of the region of interest to specify a particular joint to be measured. This is the only operator-dependent procedure in the entire measurement process. All CAJSA measurements were performed by the same operator. The CAJSA software is based on automatic edge filtering within the region of interest identifying the specified bones and their cortical edges. This was followed by calculation of the mean average and the standard deviation by the CAJSA software. The JSD measurements for each articulation and for all MCP joints (from thumb to the small finger) were given in centimetre. A total JSD-MCP (JSD-MCP_total_) was calculated.

All images and treatment regimens were blinded to the assessor.

The joint space narrowing (JSN) scoring using the modified Sharp/van der Heijde scoring depended on the smallest point in the joint space [2], whereas CAJSA-JSD measurements quantify the mean JSD in a predefined interval.

### Radiographic scoring

The radiographic scoring was performed as published by in the RAPID 1 trial using the modified total Sharp score (mTTS), including the erosion score (ES) and JSN score [11].

### Statistical analysis

We used the Mann-Whitney *U* test to compare baseline characteristics between the three treatment groups (placebo/MTX, CZP 200 mg/MTX, CZP 400 mg/MTX). To compare the radiographic scoring by mTTS and JSW as measured by CAJSA between baseline and week 52, the Wilcoxon signed-rank test was applied. Additionally, the DAS28 (erythrocyte sedimentation rate; DAS28-ESR) was used to quantify clinical remission. The study cohort was stratified in patients with clinical remission (DAS28 ≤ 2.6) and without clinical remission (DAS28 > 2.6). The differences were also compared with the Wilcoxon signed-rank test. Finally, the JSD-MCP was compared in patients with an improved Health Assessment Questionnaire Disability Index (HAQ-DI) to patients without any improvement of the HAQ-DI. The Mann-Whitney *U* test was used to compare both groups.

Further, *P* values < 0.05 were considered as statistically significant. Statistical analysis was performed by using SPSS® version 24.0 (IBM SPSS Statistics, Chicago, IL, USA), for Windows.

## Results

### Baseline characteristics (for details, see Table 1)

The post hoc analysis included 328 RA patients (265 women and 63 men) with a mean age of 51.2 ± 10.7 years and a mean disease duration of 6.0 ± 4.0 years. There were no significant differences between the groups at baseline with regard to rheumatoid factor (RF) positivity, c-reactive protein (CRP), ESR (1st hour), DAS28-ESR, health assessment questionnaire (HAQ), mTSS, ES and JSN.

**Table 1:** Baseline characteristics of the study population regarding the treatment groups (*MTX* methotrexate, *CZP* certolizumab pegol, *SD* standard deviation, *RF* rheuma factor, *CRP* C-reactive protein, *ESR* erythocyte sedimentation rate, *DAS28* Disease Activity Score 28, *HAQ-DI* Health Assessment Questionnaire Disability Index, *mTSS* modified total Sharp score, *ES* erosion score and *JSN* joint space narrowing score)

		Placebo/MTX	CZP 200 mg/MTX	CZP 400 mg/MTX
**Patient characteristics**	Number	31	149	148
	Women	26	116	123
	Men	5	33	25
	Age (in years) mean ± SD	51.1 ± 10.4	50.6 ± 11.2	51.9 ± 10.3
	Disease duration (in years), mean ± SD	6.5 ± 3.9	6.3 ± 4.3	5.6 ± 3.9
	RF positive	77.4%	80.5%	84.5%
**Disease activity status**	CRP (in mg/l), mean ± SD	21.2 ± 15.9	23.8 ± 26.7	23.5 ± 31.1
	ESR (1st hour) in mm, mean ± SD	50.4 ± 22.1	48.9 ± 24.5	46.2 ± 21.2
	DAS28-ESR, mean ± SD	6.9 ± 0.9	6.7 ± 0.8	6.8 ± 0.8
	HAQ-DI, mean ± SD	1.6 ± 0.7	1.6 ± 0.6	1.7 ± 0.6
**Radiographic status**	mTSS (Sharp units) mean ± SD	26.4 ± 32.2	32.3 ± 36.8	29.6 ± 36.1
	ES (Sharp units) mean ± SD	9.2 ± 15.6	11.2 ± 15.8	11.6 ± 17.6
	JSN (Sharp units) mean ± SD	16.7 ± 19.2	20.8 ± 23.0	17.6 ± 20.3

### Radiographic progression over 52 weeks

For the placebo/MTX-arm, a non-significant change of mTSS from 26.4 ± 32.2 Sharp units (baseline) to 27.4 ± 31.8 Sharp units (week 52) was evaluated. A similar result was observed for JSN (16.7 ± 19.2 Sharp units [baseline] to 17.8 ± 21.1 Sharp units [week 52]; *P* < 0.05) and ES (9.2 ± 15.6 Sharp units [baseline] to 9.7 ± 17.1 Sharp units [week 52]; *P* = n. s.). Regarding CZP 200 mg/MTX, a non-significant increase was revealed for mTSS (32.3 ± 36.8 Sharp units [baseline] to 32.8 ± 36.9 Sharp units [week 52]), JSN (20.8 ± 23.0 Sharp units [baseline] to 20.9 ± 21.7 Sharp units [week 52]) and ES (11.2 ± 15.8 Sharp units [baseline] to 11.6 ± 16.2 Sharp units [week 52]). The CZP 400 mg/MTX-arm showed a decrease of mTSS (29.3 ± 36.1 Sharp units [baseline] to 29.2 ± 35.8 Sharp units [week 52], *P* < n. s.) and ES (11.6 ± 17.6 Sharp units [baseline] to 11.5 ± 17.4 Sharp units [week 52], *P* < n. s.) as well as a stable JSN (17.6 ± 20.3 Sharp units [baseline] and 17.6 ± 20.4 Sharp units [week 52], *P* < n. s.).

### JSW over 52 weeks

Patients treated with placebo/MTX showed a significant joint space reduction of − 4.8% for JSD-MCP_total_ from 0.151 ± 0.028 cm (baseline) to 0.144 ± 0.033 cm (week 52). For participants who received CZP 400 mg/MTX, a non-significant change (+ 0.6%) of JSD-MCP_total_ from 0.146 ± 0.034 cm (baseline) to 0.147 ± 0.036 cm (week 52) was observed. No reduction of JSD was also evaluated for the CZP 200 mg/MTX-arm (JSD-MCP_total_: 0.147 ± 0.035 cm [baseline] to 0.147 ± 0.036 cm [week 52]).

### JSW and DAS (for details, see Tables 2 and 3 as well as Fig. 1)

Patients treated with CZP 200 mg/MTX and a DAS28 remission (DAS28 ≤ 2.6) showed a significant joint space increase of + 5.9% for JSD-MCP_total_ from 0.153 ± 0.030 cm (baseline) to 0.162 ± 0.031 cm (week 52). This was also applicable for the CZP 400 mg/MTX group and DAS28 remission (DAS28 ≤ 2.6), yielding a significant (*p* < 0.05) joint space increase of JSD-MCP_total_ + 3.3% from 0.152 ± 0.031 cm (baseline) to 0.157 ± 0.034 cm (week 52).

**Table 2:** Changes of finger JSD of MCP joints (from thumb to little finger) based on DAS28 remission in RA patients treated with CZP 200 mg plus MTX (*P < 0.01; **P = n. s.)

	Finger joint space distance	Baseline, mean (SD) in mm	Week 52, mean (SD) in mm	Difference
**DAS28 ≤ 2.6**	JSD-MCP_thumb_	0.150 ± 0.041	0.163 ± 0.032	+ 8.7%*
	JSD-MCP_index finger_	0.154 ± 0.042	0.168 ± 0.035	+ 9.1%*
	JSD-MCP_middle finger_	0.160 ± 0.040	0.164 ± 0.042	+ 2.5%*
	JSD-MCP_ring finger_	0.157 ± 0.039	0.158 ± 0.040	+ 0.6%**
	JSD-MCP_little_	0.147 ± 0.035	0.157 ± 0.037	+ 6.8%*
	**JSD-MCP**_**total**_	**0.153 ± 0.030**	**0.162 ± 0.031**	+ 5.9%*
**DAS28 > 2.6**	JSD-MCP_thumb_	0.143 ± 0.038	0.140 ± 0.040	− 2.0%**
	JSD-MCP_index finger_	0.153 ± 0.066	0.150 ± 0.067	− 1.9%*
	JSD-MCP_middle finger_	0.148 ± 0.053	0.146 ± 0.051	− 1.4%**
	JSD-MCP_ring finger_	0.146 ± 0.038	0.145 ± 0.037	− 0.6%**
	JSD-MCP_little_	0.137 ± 0.039	0.135 ± 0.042	− 1.5% **
	**JSD-MCP**_**total**_	**0.146 ± 0.037**	**0.143 ± 0.037**	− 2.1%**

**Table 3:** Changes of finger JSD of MCP (from thumb to little finger) based on DAS28 remission for RA patients treated with CZP 400 mg plus MTX (*P < 0.01; **P = n. s)

	Finger joint space distance	Baseline mean (SD) in mm	Week 52 mean (SD) in mm	Difference
**DAS28 ≤ 2.6**	JSD-MCP_thumb_	0.147 ± 0.044	0.147 ± 0.051	0%**
	JSD-MCP_index finger_	0.160 ± 0.060	0.169 ± 0.055	+ 5.6%*
	JSD-MCP_middle finger_	0.157 ± 0.038	0.162 ± 0.038	+ 3.2%*
	JSD-MCP_ring finger_	0.153 ± 0.024	0.156 ± 0.022	+ 2.0%*
	JSD-MCP_little_	0.142 ± 0.031	0.148 ± 0.033	+ 4.2%*
	**JSD-MCP**_**total**_	**0.152 ± 0.033**	**0.157 ± 0.034**	+ 3.3%*
**DAS28 > 2.6**	JSD-MCP_thumb_	0.147 ± 0.042	0.146 ± 0.043	− 0.7%**
	JSD-MCP_index finger_	0.149 ± 0.061	0.147 ± 0.063	− 1.3%**
	JSD-MCP_middle finger_	0.139 ± 0.049	0.139 ± 0.054	0%**
	JSD-MCP_ring finger_	0.147 ± 0.036	0.148 ± 0.038	+ 0.7%**
	JSD-MCP_little_	0.142 ± 0.033	0.142 ± 0.039	0%**
	**JSD-MCP**_**total**_	**0.145 ± 0.034**	**0.144 ± 0.036**	− 0.7%**

**Fig. 1:**
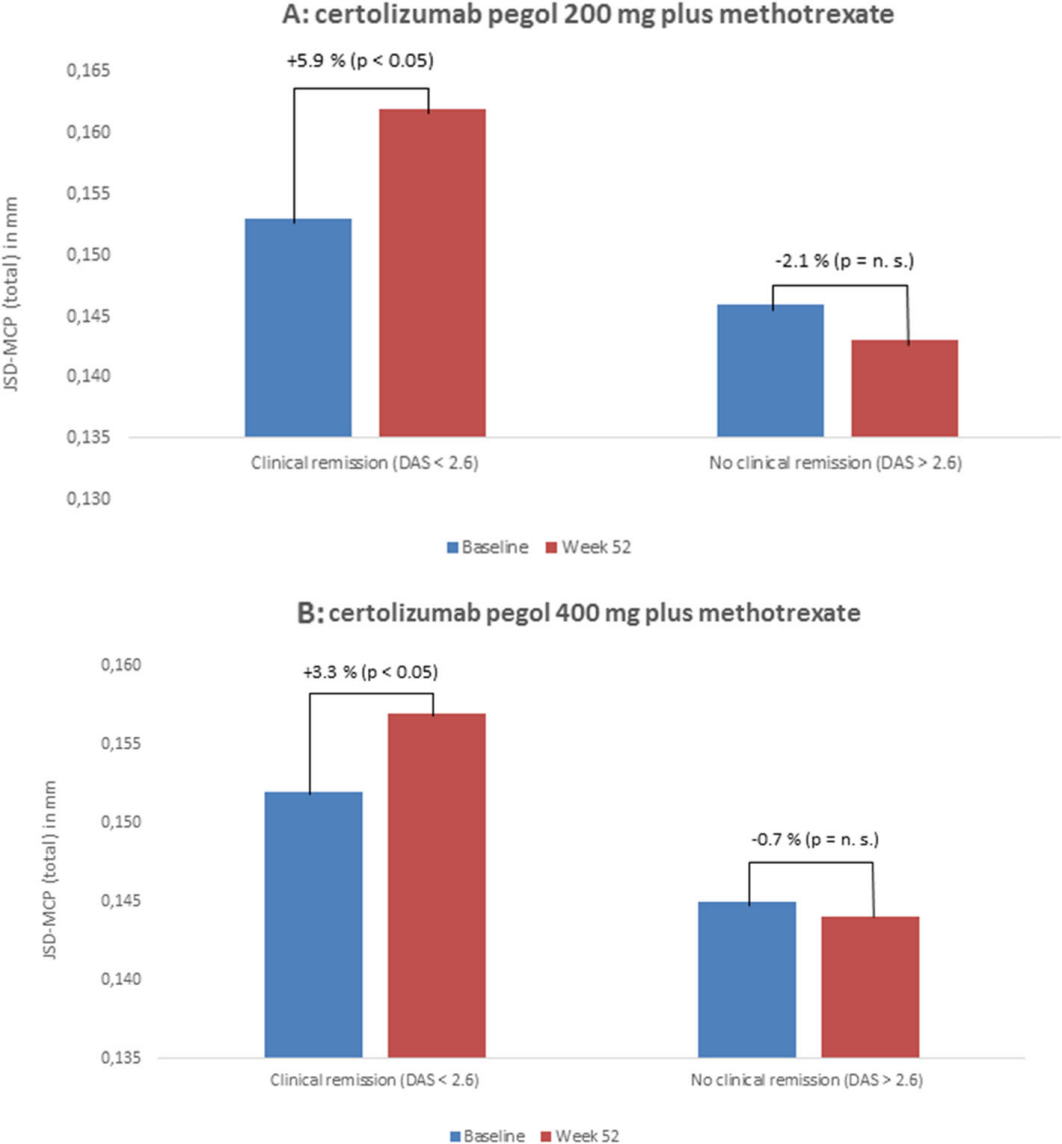
Changes of JSD-MCP_total_ between baseline and week 52 stratified for clinical remission as measured by DAS28 for **a** CZP 200 mg plus MTX and **b** CZP 400 mg plus MTX

For patients under CZP 400 mg/MTX who did not reach DAS28 remission (DAS28 > 2.6), a non-significant (*p* = n. s.) change of JSD-MCP_total_ from 0.145 ± 0.034 cm (baseline) to 0.144 ± 0.036 cm (week 52) was observed. A similar result was evaluated for the CZP 200 mg/MTX-arm (JSD-MCP_total_: 0.146 ± 0.037 cm [baseline] to 0.143 ± 0.037 cm [week 52]).

### JSW and HAQ

Patients with an improvement of the HAQ-DI over 52 weeks presented a significant wider JSW (JSD-MCP_total_: 0.148 ± 0.036 cm; + 4.2%) and CZP 400 mg/MTX (JSD-MCP_total_: 0.149 ± 0.03 cm; + 9.6%) compared to patients without an HAQ-DI improvement (CZP 200 mg/MTX: JSD-MCP_total_: 0.142 ± 0.038 cm; CZP 400 mg/MTX: JSD-MCP_total_: 0.136 ± 0.047 cm).

## Discussion

The aim of this post hoc analysis of a study subset of the RAPID 1 trial was to evaluate JSD-MCP as measured by CAJSA in RA patients treated with placebo/MTX versus CZP 200 mg/MTX and CZP 400 mg/MTX. Additionally, JSD-MCP was evaluated focusing on the impact of clinical remission.

The study revealed a non-significant change of radiographic progression as detected by mTSS, ES and JSN depending on the three treatment arms, whereas the CAJSA technique was able to quantify differences of JSW regarding the treatment with placebo/MTX versus CZP 200 mg/MTX and CZP 400 mg/MTX. In the head-to-head comparison of the Sharp JSN score and the measurement of JSD by CAJSA concerning the quantification of therapeutic effects on finger JSW, the Sharp JSN score was not able to detect changes of JSW. The detailed analysis of JSW could only be carried out with the CAJSA [9].

Further, the study presented a significant joint space reduction of JSD-MCP_total_ with − 4.8% over 52 weeks in patients only treated with placebo plus MTX. Similar results were described for MTX compared to leflunomide (LEF) using the CAJSA technique demonstrating an advanced joint space reduction [9]. Structural joint damage and radiographic progression are reflected by an increased joint space narrowing [14]. Radiographic progression is described by the change of radiographic scoring between two time points and can be detected by different established scoring techniques [15, 16] as well as the computer-aided quantification of finger JSW by CAJSA [7–9].

The treatment of RA with TNF alpha inhibitors resulted in a remarkable inhibition of radiographic progression and structural damage [17]. For example, Atsumi et al. showed a significant reduction of joint space narrowing (JSN) evaluated by the modified total Sharp score (mTSS) in RA patients treated with CZP [18]. The data of our post hoc analysis revealed no significant changes of JSD-MCP over 52 weeks for both CZP groups.

Consequently, the non-significant change of JSD-MCP is a surrogate marker for structural integrity, defined by zero radiographic progression [3, 14, 19]. With the introduction of biological and targeted synthetic DMARDs, the conventional radiographic scoring systems seem to be impaired in detecting radiographic changes and structural preservation [3, 19, 20]. The insensitivity of the conventional scoring is potentially explainable by a short treatment period of the placebo group with an escape to the treatment group following by a low radiographic progression rate in the control group [19, 20]. On the other hand, the computer-aided analysis of hand radiographs including the measurement of finger JSW seems to be more sensitive in the detection of minimal changes during radiographic progression [9, 21, 22].

Clinical remission is the treatment target in RA [23]. In our analysis, patients with DAS28 remission (DAS28 ≤ 2.6) showed a significant joint space increase of + 3.3% (CZP 200 mg/MTX) and + 3.9% (CZP 400 mg/MTX) over 52 weeks. As generally known, joint destruction in RA is comprised of cartilage and bone damage, which can be evaluated radiographically separately by the joint space narrowing (JSN) and erosion (ERO) scores, both of which are assessed separately when employing the total Sharp score (TSS) and its modification [24]. Aletaha et al. postulated that in RA patients’ cartilage, damage appears to be the more clearly associated with irreversible physical disability than bony damage [25]. The increase of JSD-MCP in RA patients with DAS28 remission could be assumed as a surrogate marker for repairing of damaged cartilage and joint. However, repair of bone erosions, defined as regression of erosion scores in RA patients, could be detected with magnetic resonance imaging (MRI) [26]. In detail, T1*ρ* mapping-based MRI strongly correlates with collagen/proteoglycan changes of the cartilage. Ku et al. reported an improved cartilage matrix health as measured by T1*ρ* mapping in RA patients under the treatment with CZP compared to MTX [27]. Consequently, healing of cartilage is potentially associated with reduced radiographic progression in RA.

Unfortunately, the current available scoring methods (e.g. Sharp score) were not able to detect reparative joint changes. The quantified increase of JSW as measured by CAJSA in combination with a clinical remission of RA represented potentially healing effects of the cartilage and confirmed the radiographic remission. In this context, this result should be verified by other imaging modalities (e.g. MRI of the cartilage) or histological studies of the cartilage.

Our study demonstrated a wider joint space in patients with an improved HAQ-DI over 52 weeks compared to patients without a HAQ-DI improvement. Aletaha et al. and Smolen et al. showed that in patients with established RA [25] and early RA [28], the cartilage damage evaluated by joint space narrowing is more associated with impairment of physical functioning than bone erosions.

Our post hoc analysis demonstrated radiographic remission with joint space widening under the treatment with CZP. To the best of our knowledge, this is the first study evaluating computer-aided measurements of finger JSW in patients treated with biologic DMARDs. Therefore, further studies are required with different substances (e.g. targeted synthetic DMARDs) to verify radiological remission.

As a potential limitation of our study, the influence of X-ray imaging parameters as well as joint gap width detection should be discussed. In this context, an experimental study revealed no significant influence of the image acquisition protocol on measurement JSD [29]. Additionally, the CAJSA-system checked the image quality and stopped the measurements if the image quality was poor and if there was a misalignment of the joints. Furthermore, CAJSA measurement of JSD is influenced by a hand rotation of more than 15° [10] which corresponded to an oblique projection [10]. In our study, we did not use oblique hand radiographs for CAJSA-JSD measurements.

## Conclusion

In conclusion, radiographic progression is detectable by conventional scoring methods and CAJSA. CAJSA allows sufficient quantification of structural integrity, reflecting the inhibition of joint space narrowing under treatment with CZP. Furthermore, clinical remission (DAS28 ≤ 2.6) was associated with an increasing joint space width which indicates radiographic remission in RA.

## Data Availability

The datasets used and/or analysed during the current study are available from the corresponding author on reasonable request.

## Abbreviations

CAJSA: Computer-aided joint space analysis
CRP: C-reactive protein
CZP: Certulizumab pegol
DAS28: Disease Activity Score 28
ES: Erosion score
ESR: Erythrocyte sedimentation rate
HAQ-DI: Health Assessment Questionnaire Disability Index
JSD: Joint space distance
JSD-MCP: Joint space distance of the metacarpal-phalangeal joint
JSN: Joint space narrowing score
JSW: Joint space width
MCP: Metacarpal-phalangeal joint
mTSS: Modified total Sharp score
n. s.: Not significant
RA: Rheumatoid arthritis

## References

[1] Smolen JS, Aletaha D, McInnes IB (2016). Rheumatoid arthritis. Lancet.

[2] van der Heijde D, Landewé R, Klareskog L, Rodriquez-Valverde V, Settas L, Pedersen R (2005). Presentation and analysis of data on radiographic outcome in clinical trials. Arthritis Rheum.

[3] Landewé RB, Connell CA, Bradley JD, Wilkinson B, Gruben D, Strengholt S (2016). Is radiographic progression in modern rheumatoid arthritis trials still a robust outcome? Experience from tofacitinib clinical trials. Arthritis Res Ther.

[4] Böttcher J, Pfeil A, Rosholm A, Petrovitch A, Seidl BE, Malich A (2005). Digital X-ray radiogrammetry combined with semi-automated analysis of joint space distances as a new diagnostic approach in rheumatoid arthritis – a cross-sectional and longitudinal study. Arthritis Rheum.

[5] Ichikawa S, Kamishima T, Sutherland K, Fukae J, Katayama K, Aoki Y (2017). Computer-based radiographic quantification of joint space narrowing progression using sequential hand radiographs: validation study in rheumatoid arthritis patients from multiple institutions. J Digit Imaging.

[6] Platten M, Kisten Y, Kälvesten J, Arnaud L, Forslind K, van Vollenhoven R (2017). Fully automated joint space width measurement and digital X-ray radiogrammetry in early RA. RMD Open.

[7] Pfeil A, Schäfer ML, Lehmann G, Seidl BE, Eidner T, Malich A (2009). Implementation of Z-scores as an age- and sex-independent parameter for estimating joint space widths in rheumatoid arthritis. J Rheumatol.

[8] Pfeil A, Renz DM, Hansch A, Kainberger F, Lehmann G, Malich A (2013). The usefulness of computer-aided joint space analysis in the assessment of rheumatoid arthritis. Joint Bone Spine.

[9] Pfeil A, Oelzner P, Bornholdt K, Hansch A, Lehmann G, Renz DM (2013). Joint damage in rheumatoid arthritis: assessment of a new scoring method. Arthritis Res Ther.

[10] Pfeil A, Hansch A, Sommerfeld J, Fröber R, Renz DM (2012). Reproducibility and influence of hand rotation on computer-aided joint space analysis. Joint Bone Spine.

[11] Keystone E, van der Heijde D, Mason D, Landewe R, van Vollenhoven R, Combe B (2008). Certolizumab pegol plus methotrexate is significantly more effective than placebo plus methotrexate in active rheumatoid arthritis: findings of a fifty-two-week, phase III, multicenter, randomized, double-blind, placebo-controlled, parallel-group study. Arthritis Rheum.

[12] Pfeil A, Nussbaum A, Renz DM, Jung C, Oelzner P, Malich A (2019). Inhibition of periarticular bone loss is associated with clinical remission and ACR70-response in rheumatoid arthritis. Rheumatol Int.

[13] van Riel PLCM (2014). The development of the Disease Activity Score (DAS) and the Disease Activity Score using 28 joint counts (DAS28). Clin Exp Rheumatol.

[14] van der Heijde D, Landewé R (2018). Should radiographic progression still be used as outcome in RA?. Clin Immunol.

[15] Mahmood S, van Tuyl L, Schoonmade LJ, Landewé R, van der Heijde D, Twisk J (2019). Systematic review of rheumatoid arthritis clinical studies: suboptimal statistical analysis of radiological data. Semin Arthritis Rheum.

[16] Salaffi F, Carotti M, Beci G, Di Carlo M, Giovagnoni A (2019). Radiographic scoring methods in rheumatoid arthritis and psoriatic arthritis. Radiol Med.

[17] Atsumi T, Yamamoto K, Takeuchi T, Yamanaka H, Ishiguro N, Tanaka Y (2016). The first double-blind, randomised, parallel-group certolizumab pegol study in methotrexate-naive early rheumatoid arthritis patients with poor prognostic factors, C-OPERA, shows inhibition of radiographic progression. Ann Rheum Dis.

[18] Atsumi T, Tanaka Y, Yamamoto K, Takeuchi T, Yamanaka H, Ishiguro N (2017). Clinical benefit of 1-year certolizumab pegol (CZP) add-on therapy to methotrexate treatment in patients with early rheumatoid arthritis was observed following CZP discontinuation: 2-year results of the C-OPERA study, a phase III randomised trial. Ann Rheum Dis.

[19] Landewé R, Strand V, van der Heijde D (2013). From inhibition of radiographic progression to maintaining structural integrity: a methodological framework for radiographic progression in rheumatoid arthritis and psoriatic arthritis clinical trials. Ann Rheum Dis.

[20] van der Heijde D, Tanaka Y, Fleischmann R, Keystone E, Kremer J, Zerbini C (2013). Tofacitinib (CP-690,550) in patients with rheumatoid arthritis receiving methotrexate: twelve-month data from a twenty-four-month phase III randomized radiographic study. Arthritis Rheum.

[21] Pfeil A, Oelzner P, Renz DM, Lehmann G, Wolf G, Böttcher J (2014). Visualisation of structural damage as a surrogate marker of radiographic progression in patients with rheumatoid arthritis. Ann Rheum Dis.

[22] Pfeil A, Oelzner P, Renz DM, Hansch A, Wolf G, Böttcher J (2015). Is there a role for digital X-ray radiogrammetry as surrogate marker for radiological progression and imaging of structural integrity in rheumatoid arthritis?. BMC Musculoskelet Disord.

[23] Smolen JS, Landewé RBM, Bijlsma JWJ, Burmester G, Dougados M, Kerschbaumer A (2020). EULAR recommendations for the management of rheumatoid arthritis with synthetic and biological disease-modifying antirheumatic drugs: 2019 update. Ann Rheum Dis.

[24] Verstappen SM, Poole AR, Ionescu M, King LE, Abrahamowicz M, Hofman DM (2006). Utrecht Rheumatoid Arthritis Cohort Study group (SRU). Radiographic joint damage in rheumatoid arthritis is associated with differences in cartilage turnover and can be predicted by serum biomarkers: an evaluation from 1 to 4 years after diagnosis. Arthritis Res Ther.

[25] Aletaha D, Funovits J, Smolen JS (2011). Physical disability in rheumatoid arthritis is associated with cartilage damage rather than bone destruction. Ann Rheum Dis.

[26] Møller Døhn U, Boonen A, Hetland ML, Hansen MS, Knudsen LS, Hansen A (2009). Erosive progression is minimal, but erosion healing rare, in patients with rheumatoid arthritis treated with adalimumab. A 1 year investigator-initiated follow-up study using high-resolution computed tomography as the primary outcome measure. Ann Rheum Dis.

[27] Ku E, Pedoia V, Tanaka M, Heilmeier U, Imboden J, Graf J (2017). Evaluating radiocarpal cartilage matrix changes 3-months after anti-TNF treatment for rheumatoid arthritis using MR T1ρ imaging. J Magn Reson Imaging.

[28] Smolen JS, van der Heijde DM, Keystone EC, van Vollenhoven RF, Goldring MB, Guérette B (2013). Association of joint space narrowing with impairment of physical function and work ability in patients with early rheumatoid arthritis: protection beyond disease control by adalimumab plus methotrexate. Ann Rheum Dis.

[29] Pfeil A, Sommerfeld J, Fröber R, Lehmann G, Malich A, Hansch A (2011). Feasibility study of semi-automated measurements of finger joint space widths. Rheumatol Int.

